# Serlogical, virulence, and molecular characterization of clinical and seafood *Vibrio parahaemolyticus* isolates

**DOI:** 10.1128/spectrum.03792-25

**Published:** 2026-05-26

**Authors:** Yan Zhang, Xiaoyun Zhang, Shuo Yuan, Quynh Huong Luu, Shuqi Lu, Quan Zhang, Quan Wang, Xiangan Han, Wei Jiang

**Affiliations:** 1 Shanghai Veterinary Research Institute, Chinese Academy of Agricultural Sciences 118161 , Shanghai, Minhang, China; 2 National Institute of Veterinary Research, Hanoi, Vietnam; Universita degli Studi di Bari Aldo Moro 544405 , Valenzano, Bari, Italy

**Keywords:** *Vibrio parahaemolyticus*, serotype, *tdh*, *trh*, T3SS, T6SS, *gyr*B

## Abstract

In this study, 42 clinical and 114 seafood isolates of *V. parahaemolyticus* from Shanghai, China, were investigated. Serotypes, Kanagawa phenomenon (KP), potential virulence factors, and phylogenetic relationship (*gyrB* gene) of these isolates were analyzed. In 156 isolates, a total of 28 serotypes were identified and only 3 predominant serotypes (O1:KUT, O3:K6, and O4:K2) were in common between clinical and seafood isolates. Of these, O1:KUT and O3:K6 were pandemic-associated. Results showed that 95.2% (40/42) clinical and 1.8% (2/114) seafood isolates were *tdh^+^and thus KP*. All clinical and seafood isolates tested positive for the enolase, MAM7, T3SS1, and T6SS2 genes. The T3SS2 gene encodes a Type III secretion system, while the T6SS1 gene encodes a Type IV secretion system. Both play an important role in the pathogenicity of *V. parahaemolyticus*. Data showed that 100% (42/42) of the clinical isolates, but only 2.6% (3/114) of the seafood isolates, tested positive for T3SS2, while 88.1% (37/42) of the clinical isolates and 21.1% (24/114) of the seafood isolates tested positive for the T6SS1 gene. This suggests that strains containing T3SS2 and T6SS1 could be highly pathogenic to humans and a public health threat. This study assists in elucidating the potential pathogenicity of *V. parahaemolyticus*, through which allele of certain virulence factors is present in the isolate genome. Thus, providing a new mechanism to facilitate monitoring of this disease during an epidemic, as well as helping to lay the foundation to prevent and control the disease.

**IMPORTANCE:**

*Vibrio parahaemolyticus* is a globally significant foodborne pathogen, with its ability to infect humans through seafood consumption often linked to specific serotypes and the presence of key virulence factors encoded in its genome. In this study, we analyzed 42 clinical isolates and 114 seafood isolates of *V. parahaemolyticus* obtained from Shanghai, China. Comparative analysis revealed a marked enrichment of several virulence-associated genes in the clinical isolates. Notably, the thermostable direct hemolysin gene (*tdh*) was detected in 95.2% of clinical isolates, but in only 1.8% of seafood isolates. Furthermore, the Type III Secretion System 2 (T3SS2) was present in all clinical isolates (42/42, 100%), whereas it was found in just 2.6% (3/114) of seafood isolates. These findings enhance our understanding of the pathogenic potential of *V. parahaemolyticus* and provide a foundation for predicting disease outbreaks through the surveillance of specific genetic markers.

## INTRODUCTION

*Vibrio parahaemolyticus* is a Gram-negative motile bacterium inhabiting marine and estuarine environments and frequently isolated from a variety of seafood (1). This bacterium has been recognized as one of the most important foodborne pathogens and possesses an enormous threat to food safety as well as human health worldwide (2, 3). It is the main contributor to acute gastroenteritis that is caused by eating contaminated or raw, under-cooked, or mishandled marine seafood (4, 5). Clinical signs of *V. parahaemolyticus* infections include vomiting, nausea, headaches, diarrhea, abdominal cramps, fever, chills, and even death (6–8). While most cases remain sporadic, several large-scale outbreaks highlight the epidemic potential of *V. parahaemolyticus* (9). In 2012, the consumption of shrimp contaminated with *V. parahaemolyticus* caused illness in 87.8% (100/114) of the passengers on a Spanish banquet cruise. Surprisingly, these isolates from this event were closely related to a hypervirulent strain isolated from an outbreak in the Pacific Northwest of the USA (10). Similarly, in 2017, 299 people were sickened by consuming food contaminated by *V. parahaemolyticus*, with 237 of those people showing severe symptoms (11). The large number of outbreaks of *V. parahaemolyticus* infections in humans emphasizes the importance of proper identification. The use of virulence factors, such as *tlh*, *toxR,* and *groEL,* as marker genes for *V. parahaemolyticus* characterization for years. But the use of more modern techniques became necessary as some of the marker genes were not consistently reliable (2). Today, investigations into *V. parahaemolyticus* outbreaks (12, 13) concentrated on serotype-based epidemiological studies, molecular phylogeny using the *gyrB* gene, transmission analyses, molecular detection of virulence factors, and the discovery of new molecular determinants. All of this contribute to the understanding of the emergence and rapid spread of this true pathogen (14, 15).

Serotyping remains a cornerstone of *V. parahaemolyticus* epidemiology and diagnostics, based on the combinatorial diversity of somatic (O) and capsular (K) antigens, which are used as the main criteria for strain categorization. Current serotyping systems recognize 13 O-antigens and 71 K-antigens (16), which are routinely used for clinical strain characterization (17). Serotype O3:K6 and its antigenic variants (serotypes O4:K68, O1:K25, and O1: KUT) are strongly linked to global epidemics (18). In recent years, clinical reports have been showing an increase in new *V. parahaemolyticu serotypes* infections. For example, an outbreak of *V. parahaemolyticus* with serotype O10:K4 occurred in Guangxi in 2020 (13). Another *V. parahaemolyticus* with serotype O4:KUT-*recA* was reported in patients from coastal areas of China. This new serotype has become the second most common after O3:K6 (19).

Virulence factors play an important role in bacterial pathogenicity. *V. parahaemolyticus* pathogenicity is complex, involving a combination of various virulence determinants in the development of illness. Two well-known virulence factors are the thermostable direct hemolysin (*tdh*), and the *tdh*-related hemolysin (*trh*), which contribute to its enterotoxicity and cytotoxicity (20, 21). Notably, the *trh* gene has been found to be linked to urease production through the regulation of the *ureC* gene (22, 23). Additionally, the Kanagawa phenomenon (KP) directly related to TDH activity is regarded as a helpful marker for differentiating pathogenic and non-pathogenic strains (24, 25).

Adherence to epithelial cells is critical for *V. parahaemolyticus* to initiate its infection and get through the initial host barrier. Studies show that interactions of the Multivalent Adhesion Molecule 7 (MAM7) with fibronectin and phosphatidic acid on the surface of host cells are required for fine adhesion (26). In addition, MAM7 enables *V. parahaemolyticus* to interact with type I collagen, promoting T3SS2-dependent host invasion (27). Furthermore, our previous study identified enolase as a potential virulence-related factor in *V. parahaemolyticus* (28).

Protein secretion systems are vital for prokaryotic survival, enabling bacteria to acquire nutrients and deliver virulence factors to facilitate infection. Many pathogens use Type III and Type VI secretion systems (T3SSs and T6SSs) to translocate effector proteins (virulence factors) from the bacterial cytosol into host cells (29). The distribution of T3SSs and T6SSs and the effectors they secrete vary among strains of the same bacterial species. *V. parahaemolyticus* possesses two sets of T3SSs (T3SS1 & T3SS2), which play an important role in effector delivery into host cells (30). T3SS1 is encoded on chromosome I and may influence how *V. parahaemolyticus* interacts with specific marine hosts, as well as how well-adapted some strains are to their environments (30, 31). T3SS2 is encoded on the *V. parahaemolyticus* chromosome II and has been associated with pathological illness in animal infection models and is considered a potential virulence marker (24, 32).

In recent years, T6SS has gained increasing attention for its role in bacterial pathogenesis, as demonstrated in *Vibrio cholerae* (33), *Francisella tularensis* (34), and *Aeromonas salmonicida* (35). For instance, T6SS-positive *Klebsiella pneumoniae* strains cause reduced survival and higher mortality in infected mice (36). In *E. coli*, T6SS has been shown to exacerbate acute liver and/or kidney injury (37). *V. parahaemolyticus* has two separate T6SS gene clusters, namely, T6SS1 and T6SS2 on chromosomes I and II, respectively. Recent studies indicated that *V. parahaemolyticus* T6SS1 and T6SS2 can mediate antibacterial competition and contribute to adhesion, biofilm formation, and antimicrobial activity (38, 39). However, whether T6SSs could be considered to be the primary predictors of strain virulence requires further investigation.

*V. parahaemolyticus* remains a leading cause of foodborne gastroenteritis worldwide, particularly in Asia (40). As the world’s largest consumer of seafood, China bears a substantial burden of *V. parahaemolyticus* infections, a concern that is especially pronounced in coastal metropolises such as Shanghai. The city’s high seafood consumption is driven by its role as a subtropical hub for international trade and a host of major global conferences. Furthermore, Shanghai functions as a critical distribution center, importing aquatic products from regions including Japan, Korea, and the USA, while supplying inland provinces across the Yangtze River Basin and throughout the country (41). With a population of approximately 24.8 million, Shanghai records a high annual incidence of *V. parahaemolyticus* infections. However, systematic comparisons of the genetic and phenotypic characteristics between clinical and seafood-derived strains in this region remain limited, which limits source tracking during outbreaks and hinders the development of risk-based surveillance strategies.

Therefore, this study aimed to genetically and serologically characterize a geographically diverse collection of *V. parahaemolyticus* strains from Shanghai, encompassing both clinical isolates and those obtained from retail seafood. We assessed serotypes, virulence-associated genes, and phylogenetic relationships to identify potential differences between the two groups. By establishing a local pathogenic profile, this work provides critical baseline data for evaluating the virulence potential of circulating strains, tracing contamination sources, and informing future surveillance and prevention strategies for *V. parahaemolyticus* infections in high-risk coastal urban settings.

## MATERIALS AND METHODS

### Sample location, isolation, and identification of *V. parahaemolyticus*

**Fig 1:**
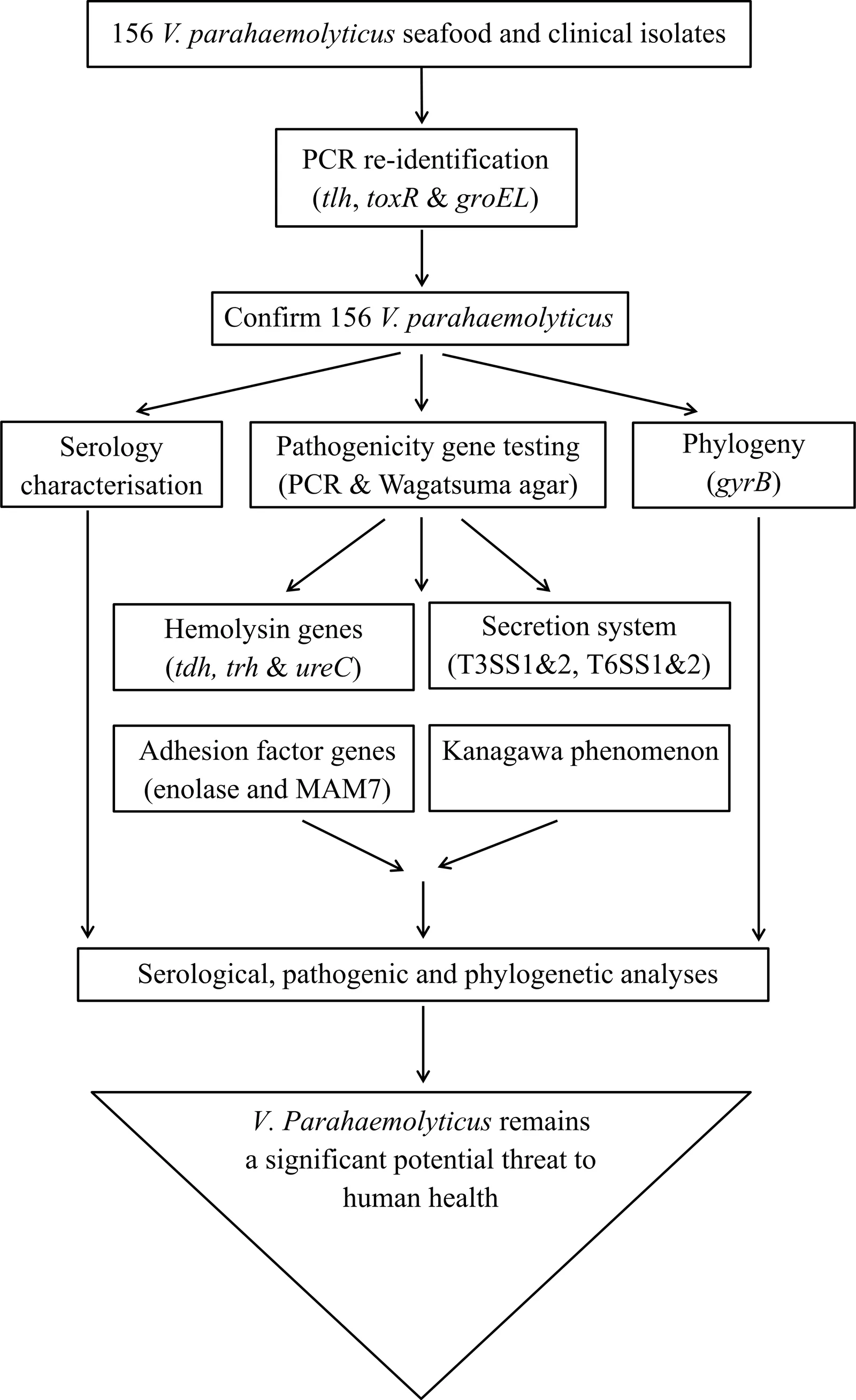
Technology roadmap for *V. parahaemolyticus* analyses.

A total of 156 *V. parahaemolyticus* isolates were used in this study. The Shanghai Center for Disease Control and Prevention (CDC) provided 42 clinical *V. parahaemolyticus* strains. The remaining 114 *V. parahaemolyticus* strains were isolated from thousands of seafood samples, including crabs, shrimps, and shellfish. These were procured from markets in Shanghai during the period from July 2006 to July 2021. Typical *V. parahaemolyticus* isolates were selected from thiosulfate-citrate-bile salts-sucrose (TCBS) plates (Oxoid, Basingstoke, UK) and chromogenic CHROMagarTM *Vibrio* (CV) agar plates (CHROMagar Microbiology, Paris, France) after an enrichment using alkaline peptone water (APW). *V. parahaemolyticus* isolates were preliminarily identified as blue-green-colored and purple-colored colonies after growth on the TCBS and CV agar plates, respectively. Tryptic soy broth (TSB), modified with 20% (vol/vol) glycerol and 3% (wt/vol) sodium chloride, was used to preserve the isolated cultures (Fig. 1).

### PCR identification of *V. parahaemolyticus*

Polymerase chain Rraction (PCR) assays were performed to detect the presence of species-specific marker genes (*tlh*, *toxR,* and *groEL*) in all 156 *V. parahaemolyticus* isolates, respectively. The primer sequences and their origins were listed in Table S1. Genomic DNA from *V. parahaemolyticus* was prepared using a DNA rapid extraction kit (UltraPure, Shanghai, China) in triplicate and stored at −20°C. Genomic DNA from *V. parahaemolyticus* strains ATCC 33,847 (*tdh+*) and ATCC 17,802 (*trh+*) served as positive controls. Genomic DNA from other *Vibrio* spp. and distilled water served as negative controls. An Eppendorf thermal cycler (Eppendorf, Germany) was used for the amplification process. The denaturation step was at 94°C for 4 min, followed by 30 cycles of 30 s at 94°C, 50 s at 55°C, and 1 min at 72°C. The PCR products were analyzed by electrophoresis using 2% agarose TBE gels. PCR products of the correct size were purified from the agarose gels using the AxyPrepTM DNA Gel Extraction kit (Axygen, Union City, USA). All purified products were then directly sequenced by Shanghai Invitrogen Biotechnology Company.

### Serology

O (lipopolysaccharide) and K (capsular) serotypes were determined using a commercially available *V. parahaemolyticus* antisera test kit (Denka Seiken, Tokyo, Japan). The fresh cultures grown on TSA plate containing 3% (wt/vol) NaCl were used for serological reactions. K-typing and O-grouping were carried out by slide agglutination following the manufacturer’s instructions. In summary, for K antigen typing, a heavy colony suspension was prepared in normal saline. A 10-μL loopful of the colony suspension was blended into one drop of each pool of K antisera and examined for agglutination. For O antigen typing, a heavy suspension was prepared in 0.5 mL saline and autoclaved at 121°C for 1.5 h. One drop of each O antiserum was mixed with a 10-μL loopful of the suspension before being tested for agglutination. A fresh cell suspension was prepared and autoclaved for 2 h before checking if the isolate did not agglutinate in any O antisera.

### Pathogenicity gene testing

All 156 *V. parahaemolyticus* isolates were tested for the presence of the hemolysin genes (*tdh* and *trh*), two adhesion-related genes (enolase and MAM7) and *ureC* gene using PCR. All *V. parahaemolyticus* strains were tested for the distribution and variation of T3SS1, T3SS2α, T3SS2β, T6SS1, and T6SS2. Representative structural and functional genes were selected based on their essential roles. The primers for these genes are described in the Appendix (Table S1).

### Kanagawa phenomenon

*V. parahaemolyticus* strains to be tested were cultured in tryptic soy broth containing 3% NaCl for 18 h and then added dropwise to Wagatsuma agar plates containing 5% rabbit erythrocytes for 24 h at 37°C. The presence of distinct β-hemolytic zones around the colonies on Wagatsuma agar plates was considered positive (42).

### Sequencing of the gyrB gene

The *gyrB* gene (Accession No. EU130502.1) of *V. parahaemolyticus* was amplified using the primers 5′-GATGATAACTCGTACAAAGT-3′ and 5′-GTCATGATGATGATGTTGT-3′. Under the predetermined conditions, the PCR procedure yielded an approximately 1,181 bp fragment. The PCR results of the *gyrB-*positive samples were electrophoresed on 2% agarose TBE gels for analysis, and the DNA of the proper size was extracted from the agarose gel using the AxyPrepTM DNA Gel Extraction kit (Axygen, Union City, USA). All purified PCR products were directly sequenced by Shanghai Invitrogen Biotechnology Company. Nucleotide sequences of *gyrB* gene fragments were uploaded to the GenBank database with accession numbers OP957446 to OP957496.

### Data analysis

Multiple sequence alignment analysis revealed 51 distinct *gyrB* sequences, all of which were used for phylogenetic analysis using the neighbor-joining (NJ) algorithm in MEGA 11.0, with bootstrap values calculated. Based on phylogenetic relationships, 16 representative clades were identified and selected for further analysis. These 16 sequences, along with an additional 46 *gyrB* sequences from various strains identified using the BLAST algorithm at NCBI (Table S2), were subjected to further phylogenetic and epidemiological analyses using Bayesian inference (BI) (43). Posterior probability (pp) values were calculated by running 2,000,000 generations. Spearman’s correlation and contingency table analysis were used to assess correlations between strains and the prevalence of certain biochemical, serotype, and virulence traits using IBM SPSS Statistics 25.0 (SPSS Inc., Chicago, IL, USA). Correlation coefficients were calculated, and *P* < 0.05 was considered statistically significant.

## RESULTS

### PCR identification of *V. parahaemolyticus*

PCR and sequencing results showed that all 156 isolates tested were positive for the presence of *toxR*, *groEL,* and *tlh* genes, confirming their identity as *V. parahaemolyticus*. A small number of negative controls yielded false-positive results in the *tlh*-based PCR assay.

### Serology

In total, 28 serotypes were identified (Table 1). Among the 42 clinical isolates, the most frequent O group was O3 (26/42, 61.9%), followed by O4 (6), O1 (5), O2 (2), O10 (2), and O5 (1). Four clinical strains did not react with K antisera and were classified as O1:KUT. Nine distinct O groups were found in seafood isolates, with O11 (34/114, 29.8%), O4 (24), O5 (16), O1 (11), O3 (10), O2 (7), O10 (6), O8 (3), and O6 (3). However, more than a third (36.8%, 42/114) of the seafood isolates did not react with K antisera.

**TABLE 1:** Distribution of hemolysin genotypes and serotypes for the 156 *V. parahaemolyticus* clinical and seafood isolates collected between 2019 and 2021 from Shanghai, China^*a*^

	Clinical isolates (*n* = 42)		Seafood isolates (*n* = 114)	
Genotype	% of total	Serotype (number of isolates)	% of total	Serotype (number of isolates)
*tdh* ^*+*^ *trh* ^*–*^	88.1 (37/42)	O1:KUT (2), O2:K1 (2)	1.8 (2/114)	O3:K6 (2)
		O3:K6 (16), O3:K1 (9)		
		O4:K8 (2), O4:K2 (3)		
		O5:K2 (1), O10:K3 (2)		
*tdh* ^*–*^ *trh* ^*+*^	4.8 (2/42)	O1:K8 (1), O1: KUT (1)	0.9 (1/114)	O1:KUT (1)
*tdh*^*+*^ *trh*^*+*^	7.1 (3/42)	O1:KUT (1), O3:K1 (1)	0	
		O4:K2 (1)		
*tdh*^*–*^ *trh* ^*–*^	0		97.4 (111/114)	O1:K4 (4), O1:K5 (5),
				O1:KUT (1), O2:K28 (4),
				O2:KUT (3), O3:K5 (2),
				O3:KUT (6), O4:K2 (12)
				O4:K6 (5), O4: KUT (7)
				O5:K4 (12), O5: KUT (4)
				O6:K6 (3), O8: KUT (3)
				O10: KUT (6), O11:K68 (4)
				O11:K3 (5), O11:K5 (4)
				O11:K7 (4), O11:K9 (6)
				O11: KUT (11)

^*a*^ KUT, Stains did not react with K antisera.

O3:K6 was the dominant serotype overall (11.5%, 18/156), followed by O4:K2 (16), O5:K4 (12), O11:KUT (11), O3:K1 (10), O4:KUT (7), O3:KUT (6), O10:KUT (6), O11:K9 (6). Six serotypes were found only in clinical isolates (O1:K8, O2:K1, O3:K1, O4:K8, O5:K2, and O10:K3), and 19 serotypes were found only in food isolates (O1:K4, O1:K5, O2:K28, O2:KUT, O3:K5, O3:KUT, O4:K6, O4:KUT, O5:K4, O5:KUT, O6:K6, O8:KUT, O10:KUT, O11:K68, O11:K3, O11:K5, O11:K7, O11:K9, and O11:KUT). There were only three serotypes (O1:KUT, O3:K6, and O4:K2) present in both clinical and food isolates. The incidence of these three serotypes was greater in clinical isolates (24/42, 57.1%) than in food isolates (16/114, 14.0%).

### Distribution of hemolysin genes and adhesion factor genes

As shown in Table 1, the most common virulence genotype (37/42, 88.1%) among clinical *V. parahaemolyticus* isolates was *tdh* positive and *trh* negative (*tdh*^+^*trh*^–^), which belong to eight serotypes (O1:KUT, O2:K1, O3:K6, O3:K1, O4:K2, O4:K8, O5:K2, and O10:K3), with O3:K6 (16/37, 43.2%) as the most common. Only 2 of 42 (4.8%) clinical isolates were *tdh* negative and *trh* positive (*tdh*^–^*trh*^+^), with serotypes O1:K8 (1) and O1:KUT (1), respectively. Three isolates (7.1%, 3/42) genotypes were positive for both *tdh* and *trh,* and they were all in clinical isolates (O1:KUT (1), O3:K1 (1) and O4:K2 (1)). Of the seafood strains, only 2 of 114 (1.8%) isolates were *tdh*^+^*trh*^−^ and they were all O3:K6 serotype. Only one of 114 (0.9%) seafood isolates was *tdh*^–^*trh^+^* (O1: KUT). The most common genotype (111/114, 97.4%) among seafood isolates was negative for both *tdh* and *trh*. No seafood isolates contained both the *tdh* and *trh* genes. In total, there were only six *trh*-positive strains (*tdh*^–^*trh* ^+^ [*n* = 3] and *tdh*^+^*trh*^+^ [*n* = 3]) were positive for the *ureC* gene. All 156 isolates were positive for both adhesion factor genes, enolase and MAM7.

### Secretion system T3SS gene testing

All strains were tested for the presence of T3SS genes specific to T3SS1, T3SS2α, and T3SS2β. Five genes associated with T3SS1 were used to screen all 156 isolates. As shown in Table 2, all 42 clinical and 114 seafood isolates harbored *vcrD1*, VP1656 (VopD1), VP1657 (VopB1), VP1686 (VopS), and VP1680 (VopQ). All isolates were retested for amplification and showed the same results. All nine gene fragments targeting T3SS2α genes were present in all of the clinical isolates with *tdh^+^trh^−^*. None of the rest (*tdh^−^trh^+^*, *tdh^+^trh^+^* and *tdh^−^trh^−^*) tested positive for any of the nine T3SS2α genes. Only one clinical isolate (*tdh^+^trh*^−^) failed to amplify VPA1362 (VopB2) but was positive for the other eight T3SS2α genes. Only 1.8% (2/114) of the seafood isolates (*tdh^+^trh^−^*) were positive for all nine of the T3SS2α genes. All four T3SS2β genes were detected in the three *tdh^−^trh^+^* (clinical/seafood = 2/1) and three *tdh^+^trh^+^* strains (clinical/seafood = 3/0) examined in this investigation. Overall, clinical isolates exhibited significantly higher detection rates than seafood isolates in terms of the detection rate of the T3SS2α and T3SS2β genes. In total, 100% clinical (88.1% [37/42] for T3SS2α*^+^* and 11.9% [5/42] for T3SS2β*^+^*) and 2.7% seafood isolates (1.8% for T3SS2α*^+^* [2/114] and 0.9% for T3SS2β*^+^* [1/114]) were positive for T3SS2. The observed association in the presence of T3SS2 system genes between clinical and seafood isolates was statistically significant (*P* < 0.01). And the presence of T3SS2α or T3SS2β genes was associated with *tdh* and *trh* (*P* < 0.01) (Table S3).

**TABLE 2:** Distribution of T3SS and T6SS genes within the 156 *V. parahaemolyticus* isolates collected between 2019 and 2021 based on isolation source and hemolysin genotype

Gene	No. of clinical isolates (*n* = 42)					No. of seafood isolates (*n* = 114)				
	*tdh*^+^ *trh*^–^ (*n* = 37)	*tdh*^–^ *trh*^+^ (*n* = 2)	*tdh*^+^ *trh*^+^ (*n* = 3)	*tdh*^–^*trh* (*n* = 0)	Total	*tdh*^+^*trh*^–^ (*n* = 2)	*tdh*^–^ *trh*^+^ (*n* = 1)	*tdh*^+^ *trh*^+^ (*n* = 0)	*tdh*^–^ *trh*^–^ (*n* = 111)	Total
T3SS1	88.1%	4.8%	7.1%	0	100%	1.8%	0.9%	0	97.4%	100%
	(37/42)	(2/42)	(3/42)	(0/42)	(42/42)	(2/114)	(1/114)	(0/114)	(111/114)	(114/114)
T3SS2α	88.1%	0	0	0	88.1%	1.8%	0	0	0	1.8%
	(37/42)	(0/42)	(0/42)	(0/42)	(37/42)	(2/114)	(0/114)	(0/114)	(0/114)	(2/114)
T3SS2β	0	4.8%	7.1%	0	11.9%	0	0.9%	0	0	0.9%
	(0/42)	(2/42)	(3/42)	(0/42)	(5/42)	(0/114)	(1/114)	(0/114)	(0/114)	(1/114)
T6SS1	81%	0	7.1%	0	88.1%	1.8%	0.9%	0	18.4%	21.1%
	(34/42)	(0/42)	(3/42)	(0/42)	(37/42)	(2/114)	(1/114)	(0/114)	(21/114)	(24/114)
T6SS2	88.1%	4.8%	7.1%	0	100%	1.8%	0.9%	0	97.4%	100%
	(37/42)	(2/42)	(3/42)	(0/42)	(42/42)	(2/114)	(1/114)	(0/114)	(111/114)	(114/114)

### Secretion system T6SS gene testing

**Fig 2:**
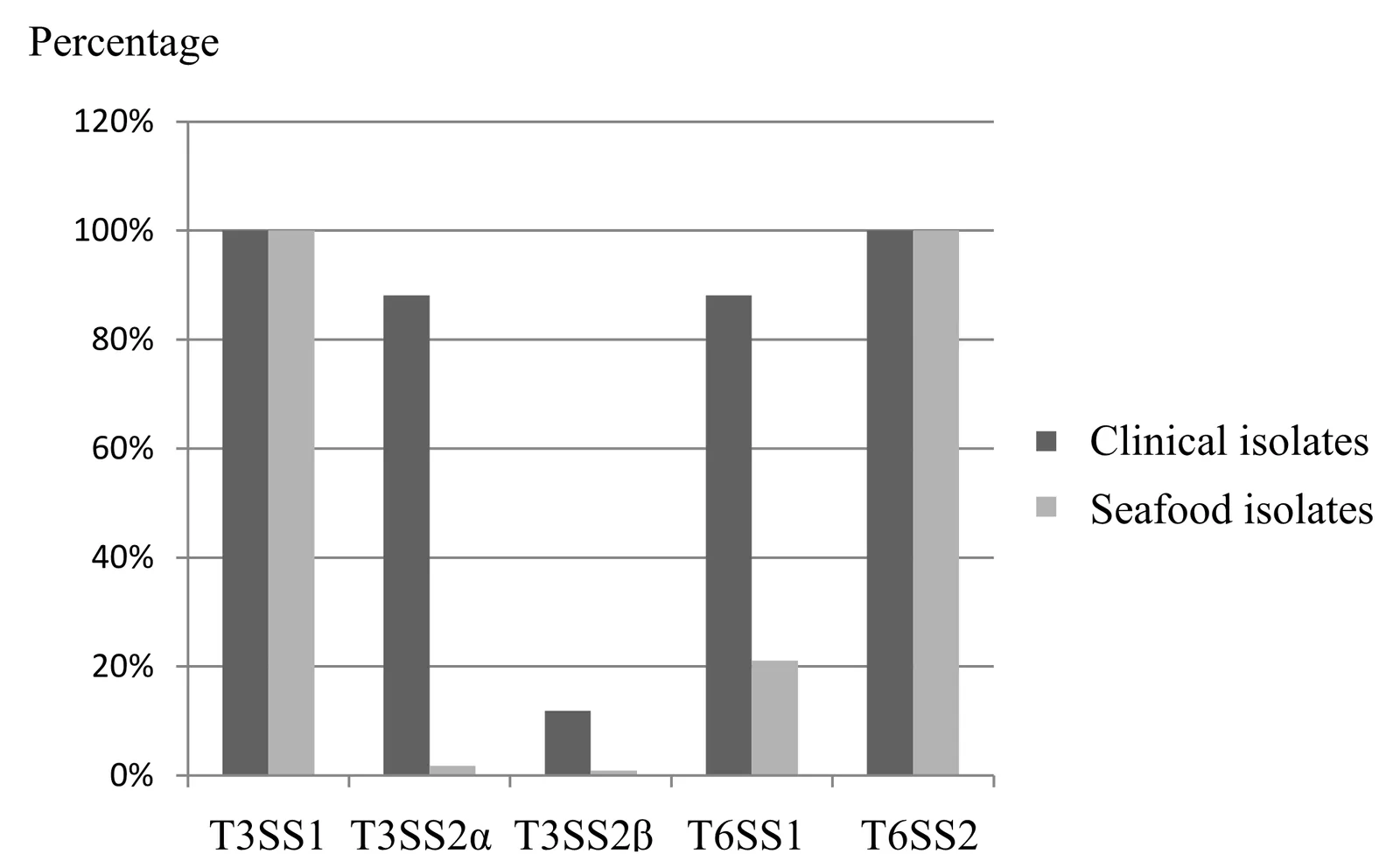
Distribution of T3SS and T6SS genes from the 156 *V. parahaemolyticus* isolated between 2019 and 2021.

All the clinical and seafood isolates contained six T6SS2 genes (Fig. 2 and Table 2). In total, 88.1% (37/42) clinical isolates either positive for *tdh* or positive for *trh* were tested positive for all 10 of the T6SS1 genes. Other five (5/42, 11.9%) clinical isolates (i.e., serotypes were O1:K8, O1:KUT, O2:K1, O5:K2, and O10:K3) either positive for *tdh* or positive for *trh* failed to amplify the 10 T6SS1 gene markers. In total, 24 of 114 (21.1%) seafood isolates amplified all 10 of the T6SS1 genes, representing various serotypes including O3:K6, O1:KUT, O4:K2, O11:K7, O5:K4, O11: KUT, O11:K68. While 12 of 114 (10.5%) seafood isolates (i.e., serotypes were O2:K28, O10:KUT, O11:K9) harbored partial T6SS1 genes, and most of them were negative for VP1391 gene target. Overall, the observed association between the clinical isolates and seafood isolates was statistically significant (*P* < 0.01) in the presence of the T6SS1 gene cluster (Table S3).

Furthermore, three T6SS effectors (VP1388, VP1415, and VPA 1263) of *V. parahaemolyticus* recently found (44) were tested for all isolates. All isolates with T6SS1*^+^* T6SS2*^+^* harbored the VP1388 gene. All three effector genes were only detected in 28.6% (12/42) of the clinical isolates, and it is interesting to note that all were positive for both T6SS1/T6SS2 and their serotypes were all O3:K6. The four remaining clinical isolates (serotype O3:K6) were only positive for VP1388 and VP1415, while failing to amplify VPA1263. Another 15 T6SS1*^+^* T6SS2*^+^* clinical isolates harbored either the VP1415 or VPA1263, with the remaining clinical isolates negative for all three effectors genes. Among the 114 seafood isolates, all three effectors were detected in only 1.8% (2/114), both of which were serotype O3:K6. A total of 24 seafood isolates were positive for both T6SS1 and T6SS2. Of these, 54.2% (13/24) were positive for either VP1415 or VPA1263, while the remaining 37.5% (9/24) were negative for all three effector genes. None of the clinical or seafood isolates that were T6SS1^–^ T6SS2^+^ amplified any of these three effector genes.

### Kanagawa phenomenon

Based on the formation of a zone of hemolysis induced by TDH on Wagatsuma agar, the Kanagawa reaction was performed as a preliminary test for the identification of virulent strains of *V. parahaemolyticus*. A positive Kanagawa reaction was detected in 34 (21.8%) strains out of a total of 156 *V. parahaemolyticus* strains, representing 25 *tdh-*positive and 9 *tdh*-negative strains. However, there were six strains containing the *tdh* gene*,* yet were KP-negative or weakly KP-positive, and nine strains were KP positive yet yielded negative results for *tdh* gene detection.

### Phylogenetic analysis of the gyrB sequence

**Fig 3:**
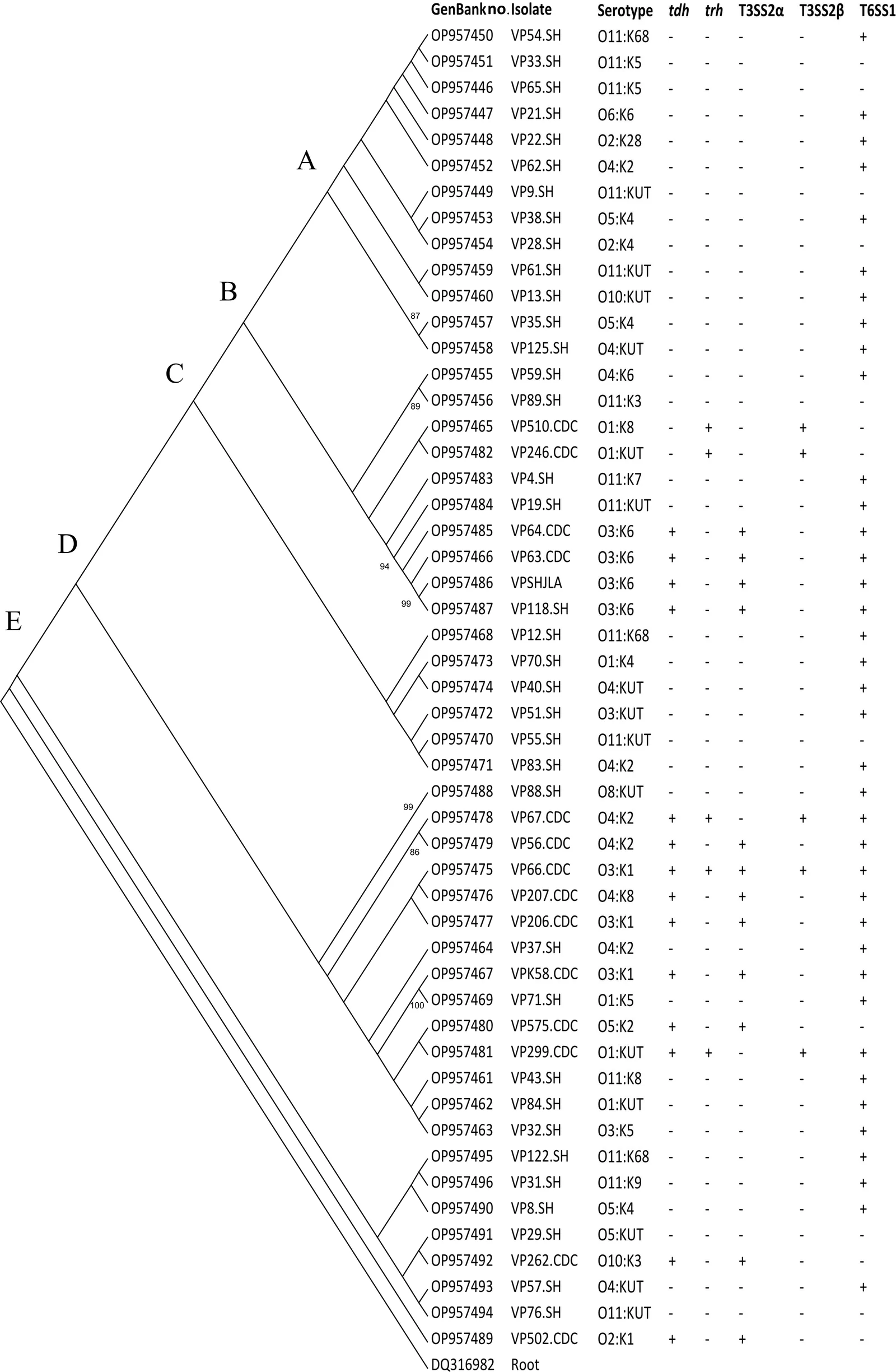
Relationships among 51 strains of *V. parahaemolyticus* in this study inferred from phylogenetic analysis of *gyrB* sequence data by neighbor-joining (NJ). Statistically significant bootstrap values are indicated on branches (i.e., bootstrap values < 85 were not shown). *V. parahaemolyticus* strains identified and characterized in the present study are indicated in bold-type. *Aeromonas hydrophila* (DQ316982) was used as root.

**Fig 4:**
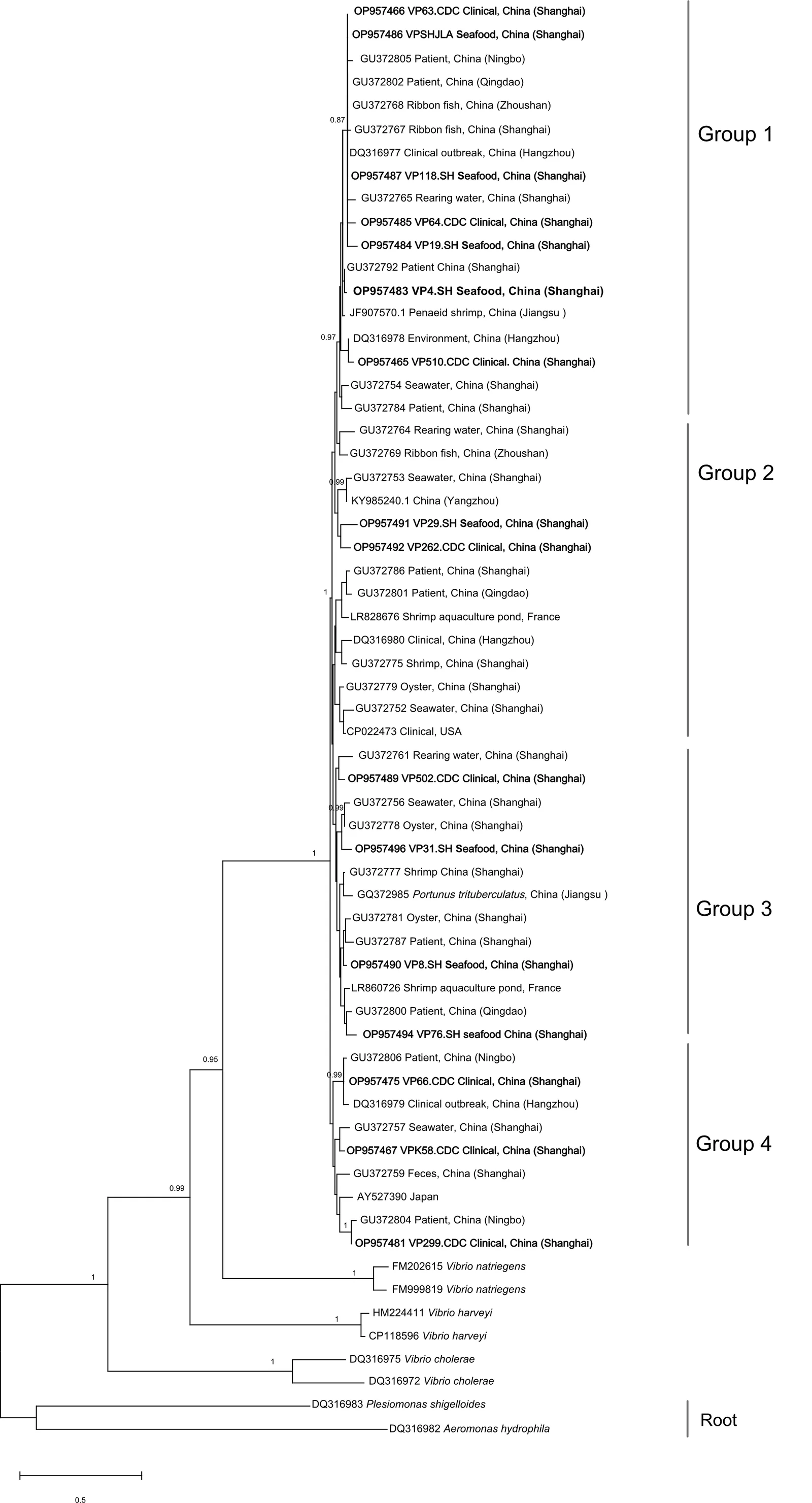
Relationships of 16 strains of *V. parahaemolyticus* in this study and 46 from NCBI and publications inferred from phylogenetic analysis of *gyrB* sequence data by Bayesian inference (BI). Statistically significant posterior probabilities (pp) are indicated on branches (i.e., bootstrap values < 0.85 were not shown). *V. parahaemolyticus* strains identified and characterized in the present study are indicated in bold-type. The scale-bar represents the number of substitutions per site. *Aeromonas hydrophila* (DQ316982) and *Plesiomonas shigelloides* (DQ316982) were used as outgroups.

In Fig. 3, the clinical and seafood isolates were distributed across different branches, representing five clades (Clades A–E). Despite being spread over five clades, there were some sequences that showed high similarity, as demonstrated by bootstrap values <85 for each clade. Clade A and Clade C only contained seafood isolates. It is notable that seafood isolate VP71.SH (Clade D) and seafood isolates VP19.SH and VP118.SH (Clade B) were closely related to clinical isolate VPK58.CDC (Clade D) as well as clinical isolates VP63.CDC, VP64.CDC, and VPSHJLA (Clade B). The phylogenetic analyses in Fig. 4 revealed four groups of *V. parahaemolyticus*. In Group 1, seafood isolates VP19.SH and VP118.SH were closely related to clinical ones (VP63.CD, VP64.CDC, and VPSHJLA) with strong statistical support (pp = 0.97). In Group 2, seafood isolate VP29.SH and clinical isolate VP262.CDC fell into a small clade, which showed close relationships, similar to the taxa found in Group 3. Although isolates in Group 4 formed a unique, observable branch, the bootstrap values showed low statistical support.

## DISCUSSION

This study aimed to detect the presence of *V. parahaemolyticus* and compare the alleles of virulence-related factors found in clinical and seafood strains isolated from Shanghai. The findings provide insights into the biology, epidemiology, and phylogeny of *V. parahaemolyticus* across these two sources. This highlights the potential public health risks within the “One Health” framework.

Accurate identification of *V. parahaemolyticus* requires innovative and more precise genetic targets to minimize the risk of both false-positive and false-negative results in PCR assays. In this study, 156 *V. parahaemolyticus* strains were positively identified using PCR amplification of three specific genes: *groEL* (45), *tlh* (46), and *toxR* (47). The PCR assays targeting either the *toxR* or *groEL* genes were consistent with high sensitivity and specificity. However, amplification of the *tlh* gene yielded many non-specific products, including homologs of closely related species such as *V. harveyi*, which can lead to false-positive results (data not shown). These findings align with previous studies reporting that the *tlh* gene alone may not be a reliable marker for *V. parahaemolyticus* due to its cross-reactivity (2). These findings suggest that the *tlh* gene may not be a consistently reliable marker for the identification of *V. parahaemolyticus*.

Clinical and foodborne isolates of *V. parahaemolyticus* exhibited signs of significant serodiversity in this study, which is consistent with the study of Chen (48). This study identified 9 distinct serotypes among clinical strains and 22 among seafood strains. Three serotypes (O1:KUT, O3:K6, and O4:K2) were common to both clinical and seafood isolates (Table 1), with O3:K6 being the most prevalent (49). Notably, our results revealed a decline in the frequency of O3:K6 strains in Shanghai. In this study, the frequency of O3:K6 from clinical (38.1%; 16/42) and seafood (1.8%; 2/114) isolates from 2019 to 2021 was lower than that observed in Shanghai a decade earlier, between 2009 and 2011. Which is supported by earlier findings (50). Nevertheless, O3:K6 remains the predominant serotype which is strongly associated with pandemic *V. parahaemolyticus* strains (51), raising our attention on clinical outbreaks in both humans and marine animals brought about through wound infections or food-borne transmissions. Additionally, the observed decline in O3:K6 is mirrored by the increases in other serotypes with potentially high pathogenicity, underscoring the need for further surveillance.

Our results showed that over 40% of seafood isolates cannot be typed with K antigen (46/114, 40.4%), while this occurred in only a small number of clinical isolates (4/42, 9.5%) (Table 1), which aligns with previous findings (31). This discrepancy may be attributed to the high recombination frequency observed in *V. parahaemolyticus* (52). Furthermore, natural transformation between *V. parahaemolyticus* and neighboring *Vibrio* species within shared ecological niches can drive the evolution of podoconjugate gene clusters (CPSgc), resulting in the presence of new K serotypes (53). Additionally, deletion events or horizontal transformation involving K antigen-related genes may further contribute to K antigen diversification (54, 55). These findings underscore the importance of ongoing surveillance and research on K serotype dynamics to better understand their epidemiological and pathogenic implications.

Thermostable direct hemolysin (TDH) and TDH-related hemolysin (TRH) are widely recognized as key virulence markers in *V. parahaemolyticus*. Studies have revealed that *tdh* or *trh* was present in more than 90% of clinical isolates but occured in fewer than 1% of the strains from seafood or environmental sources (56). Our results align with these observations, revealing that *tdh^+^* genes are predominantly found in clinical isolates (95%, 40/42), while in only 1.8% (2/114) of seafood isolates. Furthermore, our findings corroborate earlier research results, showing that all *tdh^+^* isolates also carried the *ureC* gene. Notably, the *ureC* gene was absent in *trh*^−^ strains, indicating that *tdh^+^* gene and *ureC* gene may have some unknown synergistic effect.

The activation and delivery of subsequent virulence components during infection depends on the initial contact and attachment of bacteria to a host cell. Emerging evidence suggests that MAM7-mediated adhesion during early infection stages is critical for the subsequent production or presentation of virulence components required for later infection phases (27, 57). Our previous study has demonstrated that enolase is an adhesion-related factor of *V. parahaemolyticus* with plasminogen-binding activity (28). In this study, we discovered that all *V. parahaemolyticus* strains encoded MAM7 and enolase, indicating that they are reasonably conserved among *V. parahaemolyticus*. It suggests the necessity of adhesion to epithelial cells for *V. parahaemolyticus* to cross the first host barrier although the natural host remains unknown.

In agreement with other studies, our results also showed that strains that were *trh* positive or those harboring both *trh* and *tdh* genes rarely showed hemolytic activity on Wagatsuma agar, probably due to low or no production of TDH (9, 58). However, when assessing the correlation between KP and *tdh* gene detection, we frequently observed false-positive and ambiguous results. The results revealed low sensitivity and specificity for the identification of *tdh* positive *V. parahaemolyticus*, suggesting a poor concordance within these strains between *tdh* and KP detection. These findings align with many other studies (2, 8, 59, 60), suggesting that the KP-positive reactions may stem from alternative virulence mechanisms. Thus, we strongly recommend the use of PCR assays, which has greater sensitivity and accuracy than the KP test, to identify both *tdh* and *trh* in *V. parahaemolyticus*, as we conducted in this study.

T3SS1 of *V. parahaemolyticus* has been found to be linked to host-cell cytotoxicity and was required for lethality in both a mouse intraperitoneal infection model and a lung infection model (61, 62). The gene *vcrD1* encodes a core structural component of the T3SS1 secretion apparatus, which is indispensable for its functionality (63). Additionally, VP1657 (VopB1) and VP1656 (VopD1) are essential for translocation of T3SS1 effectors into host cells (64). Previous studies have shown that T3SS1 effectors VP1680 (VopQ) and VP1686 (VopS) play key roles in immune evasion by suppressing inflammasome activation, thereby mitigating inflammasome-mediated host defenses (65). Notably, the cytotoxicity of T3SS1 is primarily attributed to the effector VP1680 (20). Our results found that all above five genes were present in all clinical and seafood strains of *V. parahaemolyticus*. Similar results were reported by Paranjpye et al. (66) who found all the clinical and environmental isolates tested were positive for both VP1686 and VP1680. Collectively, these results underscore the high conservation of the T3SS1 apparatus among *V. parahaemolyticus* strains and further corroborate its role in mediating cytotoxicity.

The gene encoding T3SS2 (α or β) is specific to highly virulent strains of *V. parahaemolyticus* (67). In contrast to the universally present T3SS1, T3SS2α and T3SS2β genes are typically associated with strains carrying *tdh^+^* and *trh^+^*, respectively (9). Our findings corroborate previous reports, demonstrating that all *tdh^+^trh^−^* isolates harbored T3SS2α, while all *trh^+^* isolates contained T3SS2β. Notably, we identified two seafood isolates (*tdh^+^trh^−^*) that possessed all the nine T3SS2α genes and one seafood isolate (*tdh^−^trh^+^*) carrying all five examined T3SS2β genes, indicating these environmental strains may possess pathogenic potential.

The virulence-associated factors of *V. parahaemolyticus* which cause certain symptoms or disorders were difficult to be categorically identified due to complicated pathophysiology. While TDH, TRH, and T3SSs are established virulence determinants that confer pathogenic status to strains harboring them (7, 68), the clinical isolation of tdh-/trh-/T3SS2-negative strains raises questions about the reliability of these markers as universal predictors of virulence (7, 68, 69). These observations underscore the existence of potentially unidentified virulence mechanisms in *V. parahaemolyticus* that warrant further investigation.

Until now, the exact role of T6SS remains unclear, but it has been implicated to be highly associated with pandemic strains (70). Both T6SS1 and T6SS2 have been shown to mediate bacterial competition (44, 71) and facilitate host cell adhesion (72), thus enhancing bacterial pathogenicity. In this study, T6SS1 was identified mainly in clinical isolates (88.1%, 37/42), while T6SS2 was found in both clinical and environmental isolates, consistent with previous studies (73, 74). Notably, 21.1% (24/114) seafood isolates harbored all 10 genes of T6SS1, with 87.5% (21/24) being *tdh^−^trh^−^* strains, suggesting that these seafood isolates may possess pathogenic potential despite lacking conventional virulence markers. The positivity rate of T6SS1 in seafood isolates (21.1%; 24/114) was significantly lower than that reported in some other studies (73.1%, tested gene *icmF1* [75]; 61.0%, *clpV1*, *hcp1*, *VgrG1*, *VipA1,* & *VipB1* [62]; 78.3%, *icmF1* [76]). However, Yu et al. (72) conducted the identification of T6SS1 (tested gene *icmF1*) and found 25.0% T6SS1-positive seafood isolates which closely aligns with our findings. The different types and numbers of genes tested for T6SS1 identification could be one of the reasons for this discrepancy, and the difference in the tested isolates may be another. While other unknown factors, such as whether strain association with different terrestrial regions is involved is another possibility, but more data are necessary to support this hypothesis.

Recent advances in comparative genomics and proteomics have identified multiple T6SS effectors (44, 77, 78). In this study, three newly identified T6SS effectors (VP1388, VP1415, and VPA1263) were selected for PCR identification (44). Molecular analysis revealed that clinical and seafood isolates with T6SS1*^+^* and T6SS2*^+^* in their genome could amplify the three effectors, confirming that these effectors were secreted by T6SSs. Notably, complete effector complement amplification occurred in only 28.5% (12/42) of clinical isolates and 1.8% (2/114) of seafood-derived strains, indicating substantial heterogeneity in effector carriage among T6SS-positive populations. Intriguingly, all triple-positive isolates belonged to the pandemic-associated O3:K6 serotype, suggesting potential serotype-specific effector conservation. Previous studies have shown that the identified T6SS effectors (VP1388, VP1415, and VPA1263) were found in the supernatant fractions of *V. parahaemolyticus* RIMD 2210633Δ*opaR*, which is O3:K6 (44, 79). However, more data need to be collected to determine whether there is an intrinsic link between the type of effectors and the serotype. Furthermore, our results showed that all isolates with T6SS1*^+^* T6SS2*^+^* could amplify VP1388, while some T6SS1*^+^* T6SS2*^+^* clinical or seafood isolates only harbored VP1415 or VPA1263. Nevertheless, they were negative for all three effector genes. The reason could be that VP1388 is encoded within the T6SS1 gene cluster, and most strains tested in this study had the complete T6SS1 sequence. Besides, VP1415 and VPA1263 may not be the required structural components of the T6SS machinery (44), and that other effectors may be remain unidentified, or regulations of T6SS translation vary corresponding to the needs of various strains (80).

Comprehensive phylogenetic reconstruction based on 51 concatenated *gyrB* sequences (Fig. 3), integrated with serotyping and virulence gene profiling, revealed significant phylogenetic clustering of clinical and seafood isolates into five distinct clades (Clades A–E) (Fig. 3). Clade B exclusively comprised pandemic-associated O3:K6 strains including clinical isolates (VP63.CDC, VP64.CDC, and VPSHJLA) and seafood isolates (VP19.SH and VP118.SH) (bootstrap values = 94–99). Similarly, in Clade D, isolates VP56.CDC and VP67.CDC clustered in one branch (bootstrap value = 86) and all exhibited the same serotype O4:K2. This indicated that taxa closely related to each other (bootstrap value > 85) may contain the same or similar serotypes although a few taxa in phylogenetic relationships showed the opposite (e.g., VP 59.SH and VP89.SH). Interestingly, we found that genetically related clinical (VP63.CDC, VP64.CDC, and VPSHJLA) and seafood isolates (VP118.SH) contained not only the same serotype O3:K6 but also the same virulence-related genes (*tdh*, T3SS2α and T6SS1), indicating that seafood isolate VP118.SH may possess high pathogenic potential and pose a public health threat. In Clade D, seafood isolate VP71.SH is closely related to clinical isolate VPK58.CDC, showing zoonotic potential. Virulence gene analyses revealed that clinical isolate VPK58.CDC had *tdh*, T3SS2α and T6SS1; however, seafood isolate VP71.SH contained T6SS1 uniquely. Similarly, we found that none of seafood isolates contained the virulence genes of *trh* and T3SS2β, but most of them (93.0%, 106/114) were T6SS1 positive and closely related to clinical isolates. This indicated that T6SS1 in seafood isolate VP71.SH may play a vital role in its pathogenicity or through interaction with other virulence molecules. Perhaps there are alternative pathways performing the similar functions as virulence genes such as *tdh, trh,* and T3SS2 may be indeterminate since the pathogenicity of *V. parahaemolyticus* isolates is complex and combinatorial (79, 81).

Bayesian phylogenetic reconstruction of 16 strains in this study and 46 strain sequence data from NCBI and their epidemiological transmissions in an international perspective were analyzed (Fig. 4). In Group 1, seafood isolates VP19.SH and VP118.SH demonstrated close phylogenetic relationships with clinical strains (VP63.CDC, VP64.CDC, and VPSHJLA), showing that these environmental isolates may possess human pathogenic potential (pp = 0.97). Interestingly, *V. parahaemolyticus* isolates (GU372765) were also found in environment such as rearing water (82), suggesting that *V. parahaemolyticus* in environment may contaminate aquatic products, then transmit this pathogen from them to humans. This commonly occurs during the summer since climate patterns are a vital risk factor to affect the cell density of *V. parahaemolyticus* in seafood (12, 83). Additionally, data in Group 1 showed that most of the isolates were located in Shanghai, Jiangsu, and Zhejiang provinces including Hangzhou, Ningbo, and Zhoushan belonging to Yangtze River Basin. These regions are seafood-rich, with many residents persisting in using raw or half-raw eating habits. Which could be one reason that *V. parahaemolyticus* outbreaks are reported almost every year (84, 85). Similarly, isolates from Groups 2–4 showed the closely related relationship between seafood isolates and clinical ones. Surprisingly, the clinical isolate in our study VP299.CDC was clustered with *V. parahaemolyticus* isolates from Japan, raising concerns about cross-border transmission. Japan is an importer of seafood from China, and Shanghai has the largest shipping ports, making *V. parahaemolyticus* global transmission reasonable. The overall results of this study emphasize the importance to keep investigating this food-borne microbe in marine products, humans, and aquatic environments worldwide, as well as the necessity of more epidemiological metadata and genetic data along with rich pathogenic information worldwide used for discussion of *V. parahaemolyticus* in a broad perspective (81).

*V. parahaemolyticus* remains a critical pathogen responsible for severe seafood-borne gastroenteric disease and even death in humans. Herein, 42 clinical and 114 seafood isolates of *V. parahaemolyticus* were identified, and their serotypes, KP multiple pathogenic factors, and phylogenetic relationships were analyzed. A high serodiversity of *V. parahaemolyticus* was found among clinical and seafood isolates, and two pandemic serotypes were in common between clinical and seafood isolates. T3SS2 and T6SS1 demonstrated strong correlations with the pathogenicity of *V. parahaemolyticus*, reinforcing their roles as critical virulence systems. Phylogenetic analyses revealed close genetic relationships between select seafood and clinical isolates, including shared serotypes and virulent gene profiles, posing a public health threat. These findings emphasize that *V. parahaemolyticus* continues to represent a significant threat to human health. The persistence of virulent strains in environmental reservoirs, coupled with their evolving genetic diversity, necessitates sustained surveillance, improved diagnostic strategies, and deeper investigations into its pathogenicity mechanisms to mitigate future outbreaks.

Despite the new insights of *V. parahaemolyticus* strains presented here, it is important to acknowledge its limitations and the challenges they posed. (i) The sources and numbers of samples studied here were limited. Future studies should incorporate more diverse clinical specimens and environmental samples (e.g., seawater, feces, and rearing water) from multiple geographic regions to enhance our epidemiological understanding of *V. parahaemolyticus* transmission dynamics; (ii) the absence of whole genome sequencing (WGS) data represents a significant limitation, largely due to the early collection period of these samples (beginning in 2006) when WGS was not yet routine. Given the dramatic advances in sequencing technologies—including reduced costs and increased accessibility—we strongly recommend implementation of WGS for future studies to enable comprehensive phylogenetic and virulence determinant analyses; (iii) the extended sample collection period (spanning over a decade) may have allowed for significant bacterial evolution. To better capture contemporary strain characteristics, future research should employ more frequent sampling intervals within shorter time frames. In summary, more efforts on investigations of a large amount of *V. parahaemolyticus* strains from both clinical and environmental sources in different locations in China using genome sequencing technique will facilitate the better understanding of the pathogenesis of *V. parahaemolyticus* and provide data in support of prevention and control technology against the leading seafood-borne pathogen worldwide.

## Data Availability

Nucleotide sequences of *gyrB* gene fragments were uploaded to the GenBank database (https://www.ncbi.nlm.nih.gov/) with accession numbers OP957446–OP957496.

## SUPPLEMENTAL MATERIAL

Table S1 spectrum.03792-25-s0001.doc Primer and probe sequences utilized in this study.

Table S2 spectrum.03792-25-s0002.docx GenBank accession numbers of all gyrB DNA sequences used for phylogenetic analysis (Fig. 2 and 3) and associated information.

Table S3 spectrum.03792-25-s0003.docx Correlation analyses between the source of the isolate (clinical versus seafood) and the virulence traits.

ASM does not own the copyrights to Supplemental Material that may be linked to, or accessed through, an article. The authors have granted ASM a non-exclusive, world-wide license to publish the Supplemental Material files. Please contact the corresponding author directly for reuse.
