# Supplementary material for: Serlogical, virulence, and molecular characterization of clinical and seafood Vibrio parahaemolyticus isolates
Source: Microbiol Spectr. 2026 May 26;14(7):e03792-25. doi: 10.1128/spectrum.03792-25 (PMC13339958; doi:10.1128/spectrum.03792-25)
Supplement: Table S1 — Primer and probe sequences utilized in this study. [file spectrum.03792-25-s0001.doc]

**Supplementary Table S1.** Primer and probe sequences utilized in this study.

| **Target**  **gene** | **Primer sequence (5' 3')** | | **Size**  **(bp)** | **Reference or source** |
| --- | --- | --- | --- | --- |
| ***V. parahaemolyticus* species-specific marker** | | | | |
| *tlh* | F | aaagcggattatgcagaagcactg | 450 | Jaime, et al. (2006) |
|  | R | GCTACTTTCTAGCATTTTCTCTGC |  |  |
| *toxR* | F | GTCTTCTGACGCAATCGTTG | 368 | Jaime, et al. (2006) |
|  | R | ATACGAGTGGTTGCTGTCATG |  |  |
| *groEL* | F | AGGTCAGGCTAAGCGCGTAAGC | 510 | Hossain, et al. (2011) |
|  | R | GTCACCGTATTCACCCGTCGCT |  |  |
| **Hemolysin genes associated with virulence** | | | | |
| *tdh* | F | GTAAAGGTCTCTGACTTTTGGAC | 269 | Jaime, et al. (2006) |
|  | R | TGGAATAGAACCTTCATCTTCACC |  |  |
| *trh* | F | TTGGCTTCGATATTTTCAGTATCT | 500 | Jaime, et al. (2006) |
|  | R | CATAACAAACATATGCCCATTTCCG |  |  |
| *ureC* | F | CTAATGCTACAACAGTCACC | 1040 | this study |
|  | R | CGACAACCTTTTACCTGACC |  |  |
| **Adhesion factors** | | | | |
| *MAM7* | F | TTACTGAGTCTGGGTTGAGC | 545 | this study |
|  | R | CGTGCCGTCGGTTTCTAG |  |  |
| *enolase* | F | ATGTCTAAGATCGTTAAAGTT | 1320 | this study |
|  | R | TTAAGCTTGGCCTTTAACT |  |  |
| **T3SS1** |  | | | |
| *vcrD1* | F | ACATCACGCGCTGCTTACCCCACT | 356 | this study |
|  | R | TCTCGCCCACGTTCGCCAATCA |  |  |
| VP1656 (*vopD1****)*** | F | ACACTGTCGGGATAGATGCG | 470 | this study |
|  | R | AGCGGGTAAAGAAATTAAGACC |  |  |
| VP1657 (*vopB1*) | F | CAGCGGCTTCTGATTCCT | 394 | this study |
|  | R | TCTACTGCGACGCCTTCT |  |  |
| VP1680 (*vopQ*) | F | ACCGTTCGCCGTAGATTC | 684 | this study |
|  | R | GGTGTAGGCTTGCTCGTC |  |  |
| VP1686 (*vopS*) | F | TTTGGTTGATGCGTACTTC | 335 | this study |
|  | R | TGACAACGAACAGGGACA |  |  |
| **T3SS2α** |  |  |  |  |
| VPA1355 (*vcrD2*) | F | CTGAACCAGGTGTAGTGA | 499 | this study |
|  | R | AGTGTAGTTTGTACCGAAA |  |  |
| VPA1321 (*vopC*) | F | GAGTTGGTTTCGCAGGTT | 575 | this study |
|  | R | GGTACGCCTCTTGGACAG |  |  |
| VPA1327（*vopT*） | F | CATCAAGTAAGGCAGGCACA | 256 | this study |
|  | R | TCAACCTCACGAGACCCAGA |  |  |
| VPA1335 (*vscS2*) | F | ATGTAACGGCGGCTAGCTTA | 174 | Noriea III, et al. (2010) |
|  | R | CAAACTGTGTCAGTAGCACCA |  |  |
| VPA1339 (*vscC2*） | F | GATTCGCGGAACTCAAGAAG | 250 | Noriea III, et al. (2010) |
|  | R | CTTGTCCGAGATCAACGTCA |  |  |
| VPA1346 (*vopA*) | F | CATTGTATAGCGGTGGACTG | 334 | this study |
|  | R | GCGGCAATAAACTATCAGCA |  |  |
| VPA1357 (*vopV*) | F | TCAGACGATGAGCAGGTTT | 787 | this study |
|  | R | GCTGTATTGGCACCATTTA |  |  |
| VPA1361 (*vopD2*) | F | ACCGTGTTGTATTTGGCAGTA | 309 | this study |
|  | R | TCGCTCGCAAGTGATGAG |  |  |
| VPA1362 (*vopB2*) | F | CTGCAGGTATCGCATCTTCA | 343 | Okada, et al. (2009) |
|  | R | TTAGAACCAACCGACGAAGC |  |  |
| **T3SS2β** |  |  |  |  |
| *vscC2* | F | GTACTTTGCTGTCTAACC | 1400 | Okada, et al. (2009) |
|  | R | CTTACTCTTAACTTCCGACG |  |  |
| *vopB2* | F | GAGCCTGTTGCTCTATGGAGCCAGG | 942 | Okada, et al. (2009) |
|  | R | CGACACAGAACGCAATGCTTGCTCG |  |  |
| *vopC* | F | AACCAACTTGCGACTAAATC | 594 | Okada, et al. (2009) |
|  | R | TCCCGACAGTTTTTCTGCAC |  |  |
| *vscS2* | F | TTGATGTTGTTTCGGCTAGC | 224 | Okada, et al. (2009) |
|  | R | CCACCGCCGAACTCGGCTAACAAG |  |  |
| **T6SS1** |  | | | |
| VP1402 (*vipA1*) | F | ATGTCACGTGACGGCTCGGT  TTACTCTTCTTTCGCGTCT | 507 | this study |
|  | R |  |  |  |
| VP1403 (*vipB1*) | F | TGCGATTCTTCACAACGA  GAGCGATAAAGCCCTCTT | 797 | this study |
|  | R |  |  |  |
| VP1393 (*hcp1*) | F | atgccaactccagc  ttaagcttcgcgtgga | 519 | this study |
|  | R |  |  |  |
| VP1394 (*vgrG1*) | F | CACAGGGCAAGACCCACAAG  TCAGGCAACGAGTACGGAGG | 420 | this study |
|  | R |  |  |  |
| VP1408 (*icmF1*) | F | TGCTGGCATCCGTTGTCTTA  CCAACAATGAGCGACGAGTT | 582 | this study |
|  | R |  |  |  |
| VP1414 (*dotU1*) | F | CGCCTGACTTGTCTCGTA  ATCGCACTGATGCCTAAA | 637 | this study |
|  | R |  |  |  |
| VP1392 (*clpV1*) | F | ACACGGTATCTCGCCTAATC  TCAGGGACAACTTGTGATGG | 468 | this study |
|  | R |  |  |  |
| VP1404 (*gp25*) | F | CTCCCACAAGCATTTGAT | 297 | this study |
|  | R | GACATTGGAACTTTACCG |  |  |
| VP1391 (*sigma54*) | F | AACCATTGGGCTGTTAGTCT  CGCAGTAACTTCGCTTGTAG | 555 | this study |
|  | R |  |  |  |
| VP1411  (*fha1*) | F | GGCGGTCGTAGAAGAGGAAG  TTGAGGCTGTTGAGGTTGGT | 415 | this study |
|  | R |  |  |  |
| VP1415 | F | TCATTTCAGCCTCACCAACG | 448 | this study |
|  | R | CTCGGCATTTCAACAGCATC |  |  |
| VPA1263 | F | CACTTGCGGTAGGTGATGAAA | 205 | this study |
|  | R | AGGATTACCACATCGCCTTCTA |  |  |
| VP1388 | F | AGGCTCTTTGAATGGCTCTG | 250 | this study |
|  | R | CTCGCTCAATCGTTTACTGTTAT |  |  |
| **T6SS2** |  | | | |
| VPA1035 (*vipA2*) | F | GCCTCGTGTTCACATTAC  CATCAAGTCTCGCAGTTT | 355 | this study |
|  | R |  |  |  |
| VPA1033 (*vipB2*) | F | CGCCATAGCATTGTGAGC  TTGGAGCATTGAGGTGAG | 1014 | this study |
|  | R |  |  |  |
| VPA1027 (*hcp2*) | F | ATTGGAGTGTTGGTCGTG  ATCGCATCAGTCAGTTCG | 226 | this study |
|  | R |  |  |  |
| VPA1026 (*vgrG2*) | F | AAAACCAGCCTCACAACC  TCGCCATTCACAAACTCTAC | 627 | this study |
|  | R |  |  |  |
| VPA1039 (*icmF2*) | F | ACAAAGAAGCCGCCAGTG  TCGCTAAAGGCAACCTCC | 945 | this study |
|  | R |  |  |  |
| VPA1028 (*clpV2*) | F | GAGGCAGGCGTAGGTAAA  TGTCTTCGCTCCCATAAA | 1207 | this study |
|  | R |  |  |  |

F=forward primer; R=reverse primer; *tlh*, thermolabile hemolysin; *toxR*, *groEL*, *tdh*, thermostable direct hemolysin gene; *trh,* TDH-related hemolysin gene; T3SS, type III secretion system; T6SS, type VI secretion systems.

**References**

Hossain, M. T., Kim, E. Y., Kim, Y. R., Kim, D. G., & Kong, I. S. (2011). Application of *groEL* gene for the species-specific detection of *Vibrio parahaemolyticus* by PCR. *Letters in Applied Microbiology, 54*(1), 67-72.

Jaime, M. U., Antonio, L. L., Alejandro, V. F., Jacobo De, D., & Oscar, G. M. (2006). Differences in the API 20E biochemical patterns of clinical and environmental *Vibrio parahaemolyticus* isolates. *FEMS Microbiology Letters, 255*(2006), 75-81.

Noriea III, N. F., Johnson, C. N., Griffitt, K. J., & Grimes, D. J. (2010). Distribution of type III secretion systems in *Vibrio parahaemolyticus* from the northern Gulf of Mexico. *Journal of Applied Microbiology, 109*(3), 953–962.

Okada, N., Iida, T., Park, K. S., Goto, N., Yasunaga, T., Hiyoshi, H., et al. (2009). Identification and characterization of a novel type III secretion system in *trh*-positive *Vibrio parahaemolyticus* strain TH3996 reveal genetic lineage and diversity of pathogenic machinery beyond the species level. *Infection and Immunity, 77*(2), 904-913.
