# Supplementary material for: Serlogical, virulence, and molecular characterization of clinical and seafood Vibrio parahaemolyticus isolates
Source: Microbiol Spectr. 2026 May 26;14(7):e03792-25. doi: 10.1128/spectrum.03792-25 (PMC13339958; doi:10.1128/spectrum.03792-25)
Supplement: Table S2 — GenBank accession numbers of all gyrB DNA sequences used for phylogenetic analysis (Fig. 2 and 3) and associated information. [file spectrum.03792-25-s0002.docx]

**Supplementary Table S2**. GenBank accession numbers of all *gyrB* DNA sequences used for phylogenetic analysis (Figs. 2&3) and associated information. Included here are sequences of (i) 44 *Vibrio* spps., one *Aeromonas hydrophila* (DQ316982, root) and one *Plesiomonas shigelloides* (DQ316983, root) from NCBI or published literature; (ii) 51 sequences of *Vibrio parahaemolyticus* identified/defined in the present study.

| **GenBank no.** | **length (*gyrB*)** | **Serotype** | **Source of isolates** | **Country** | **City** |
| --- | --- | --- | --- | --- | --- |
| AY527390 | 1123 | NA | NA | Japan | NA |
| CP022473 | 2418 | NA | NA | USA | NA |
| CP118596 | 1186 | NA | NA | NA | NA |
| DQ316972 | 1265 | O139 | Clinical | China | Hangzhou |
| DQ316975 | 1156 | O1:KUT | Clinical | China | Hangzhou |
| DQ316977 | 1160 | O3:K6 | Clinical outbreak | China | Hangzhou |
| DQ316978 | 1144 | O1:KUT | Environment | China | Hangzhou |
| DQ316979 | 1155 | O4:K8 | Clinical outbreak | China | Hangzhou |
| DQ316980 | 1156 | NA | Clinical | China | Hangzhou |
| DQ316982 | 1168 | NA | Clinical | China | Hangzhou |
| DQ316982 | 1168 | NA | Clinical | China | Hangzhou |
| DQ316983 | 1148 | NA | Clinical | China | Hangzhou |
| FM202615 | 1050 | NA | NA | NA | NA |
| FM999819 | 1051 | NA | NA | NA | NA |
| GQ372985 | 1191 | NA | *Portunus trituberculatus* | China | Jiangsu |
| GU372752 | 1181 | O11:ND | Seawater | China | Shanghai |
| GU372753 | 1181 | O1:ND | Seawater | China | Shanghai |
| GU372754 | 1181 | O1:ND | Seawater | China | Shanghai |
| GU372756 | 1181 | O5:ND | Seawater | China | Shanghai |
| GU372757 | 1181 | O5:ND | Seawater | China | Shanghai |
| GU372759 | 1181 | O5:ND | Feces | China | Shanghai |
| GU372761 | 1181 | O2:ND | Rearing water | China | Shanghai |
| GU372764 | 1181 | O4:ND | Rearing water | China | Shanghai |
| GU372765 | 1181 | O6:ND | Rearing water | China | Shanghai |
| GU372767 | 1181 | O1:ND | Ribbon fish | China | Shanghai |
| GU372768 | 1181 | O3:K6 | Ribbon fish | China | Zhoushan |
| GU372769 | 1181 | O4:K8 | Ribbon fish | China | Zhoushan |
| GU372775 | 1181 | O3:ND | Shrimp | China | Shanghai |
| GU372777 | 1181 | O5:ND | Shrimp | China | Shanghai |
| GU372778 | 1181 | O5:ND | Oyster | China | Shanghai |
| GU372779 | 1181 | O1:ND | Oyster | China | Shanghai |
| GU372781 | 1181 | O3:ND | Oyster | China | Shanghai |
| GU372784 | 1181 | O4:ND | Patient | China | Shanghai |
| GU372786 | 1181 | O3:K6 | Patient | China | Shanghai |
| GU372787 | 1181 | O3:K6 | Patient | China | Shanghai |
| GU372792 | 1181 | O4:ND | Patient | China | Shanghai |
| GU372800 | 1181 | O4:K8 | Patient | China | Qingdao |
| GU372801 | 1181 | O3:K6 | Patient | China | Qingdao |
| GU372802 | 1181 | O3:K6 | Patient | China | Qingdao |
| GU372804 | 1181 | O1:K25 | Patient | China | Ningbo |
| GU372805 | 1181 | O3:K6 | Patient | China | Ningbo |
| GU372806 | 1181 | O4:ND | Patient | China | Ningbo |
| HM224411 | 1186 | NA | NA | NA | NA |
| JF907570.1 | 1139 | NA | Penaeid shrimp | China | Jiangsu |
| KY985240.1 | 1162 | NA | NA | China | Yangzhou |
| LR828676 | 2418 | NA | Shrimp aquaculture pond | France | NA |
| LR860726 | 2418 | NA | Shrimp aquaculture pond | France | NA |
| OP957446 | 1070 | O11:K5 | Seafood | China | Shanghai |
| OP957447 | 1073 | O6:K6 | Seafood | China | Shanghai |
| OP957448 | 1087 | O2:K28 | Seafood | China | Shanghai |
| OP957449 | 1067 | O11:KUT | Seafood | China | Shanghai |
| OP957450 | 1069 | O11:K68 | Seafood | China | Shanghai |
| OP957451 | 1075 | O11:K5 | Seafood | China | Shanghai |
| OP957452 | 1073 | O4:K2 | Seafood | China | Shanghai |
| OP957453 | 1076 | O5:K4 | Seafood | China | Shanghai |
| OP957454 | 1084 | O2:K4 | Seafood | China | Shanghai |
| OP957455 | 1071 | O4:K6 | Seafood | China | Shanghai |
| OP957456 | 1076 | O11:K3 | Seafood | China | Shanghai |
| OP957457 | 1069 | O5:K4 | Seafood | China | Shanghai |
| OP957458 | 1046 | O4:KUT | Seafood | China | Shanghai |
| OP957459 | 1085 | O11:KUT | Seafood | China | Shanghai |
| OP957460 | 1086 | O10:KUT | Seafood | China | Shanghai |
| OP957461 | 1068 | O11:K8 | Seafood | China | Shanghai |
| OP957462 | 1084 | O1:KUT | Seafood | China | Shanghai |
| OP957463 | 1082 | O3:K5 | Seafood | China | Shanghai |
| OP957464 | 1087 | O4:K2 | Seafood | China | Shanghai |
| OP957465 | 1075 | O1:K8 | Clinical | China | Shanghai |
| OP957466 | 1071 | O3:K6 | Clinical | China | Shanghai |
| OP957467 | 1071 | O3:K1 | Clinical | China | Shanghai |
| OP957468 | 1061 | O11:K68 | Seafood | China | Shanghai |
| OP957469 | 1069 | O1:K5 | Seafood | China | Shanghai |
| OP957470 | 1025 | O11:KUT | Seafood | China | Shanghai |
| OP957471 | 1014 | O4:K2 | Seafood | China | Shanghai |
| OP957472 | 1020 | O3:KUT | Seafood | China | Shanghai |
| OP957473 | 1042 | O1:K4 | Seafood | China | Shanghai |
| OP957474 | 1049 | O4:KUT | Seafood | China | Shanghai |
| OP957475 | 1079 | O3:K1 | Clinical | China | Shanghai |
| OP957476 | 1051 | O4:K8 | Clinical | China | Shanghai |
| OP957477 | 1076 | O3:K1 | Clinical | China | Shanghai |
| OP957478 | 1094 | O4:K2 | Clinical | China | Shanghai |
| OP957479 | 1095 | O4:K2 | Clinical | China | Shanghai |
| OP957480 | 1076 | O5:K2 | Clinical | China | Shanghai |
| OP957481 | 1091 | O1:KUT | Clinical | China | Shanghai |
| OP957482 | 1092 | O1:KUT | Clinical | China | Shanghai |
| OP957483 | 1094 | O11:K7 | Seafood | China | Shanghai |
| OP957484 | 1081 | O11:KUT | Seafood | China | Shanghai |
| OP957485 | 1085 | O3:K6 | Clinical | China | Shanghai |
| OP957486 | 1181 | O3:K6 | Seafood | China | Shanghai |
| OP957487 | 1076 | O3:K6 | Seafood | China | Shanghai |
| OP957488 | 1079 | O8:KUT | Seafood | China | Shanghai |
| OP957489 | 1095 | O2:K1 | Clinical | China | Shanghai |
| OP957490 | 1094 | O5:K4 | Seafood | China | Shanghai |
| OP957491 | 1094 | O5:KUT | Seafood | China | Shanghai |
| OP957492 | 1082 | O10:K3 | Clinical | China | Shanghai |
| OP957493 | 1082 | O4:KUT | Seafood | China | Shanghai |
| OP957494 | 1079 | O11:KUT | Seafood | China | Shanghai |
| OP957495 | 1054 | O11:K68 | Seafood | China | Shanghai |
| OP957496 | 1095 | O11:K9 | Seafood | China | Shanghai |

ND = Not detected. NA = Not available. KUT= Stains did not react with K antisera.
