# Supplementary material for: Serlogical, virulence, and molecular characterization of clinical and seafood Vibrio parahaemolyticus isolates
Source: Microbiol Spectr. 2026 May 26;14(7):e03792-25. doi: 10.1128/spectrum.03792-25 (PMC13339958; doi:10.1128/spectrum.03792-25)
Supplement: Table S3 — Correlation analyses between the source of the isolate (clinical versus seafood) and the virulence traits. [file spectrum.03792-25-s0003.docx]

**Supplementary Table S3.** Correlation analyses between the source of the isolate (clinical versus seafood) and the virulence traits.

|  | | Strain | T3SS2α | T3SS2β | T6SS1 | *tdh* +  *tdh* – | *tdh* –  *trh* + | *tdh* +  *trh* + | T3SS1 | T6SS2 |
| --- | --- | --- | --- | --- | --- | --- | --- | --- | --- | --- |
| Strain | Correlation Coefficient |  | .884^**^ | .254^**^ | .609^**^ | .884^**^ | .125 | .231^**^ | - | - |
|  | Sig. (2-tailed) |  | .000 | .001 | .000 | .000 | .119 | .004 | - | - |
| T3SS2α | Correlation Coefficient | .884^**^ |  | .346^**^ | .721^**^ | 1.000^**^ | .243^**^ | .243^**^ | - | - |
|  | Sig. (2-tailed) | .000 |  | .000 | .000 | - | .002 | .002 | - | - |
| T3SS2β | Correlation Coefficient | .254^**^ | .346^**^ |  | .250^**^ | .346^**^ | .700^**^ | .700^**^ | - | - |
|  | Sig. (2-tailed) | .001 | .000 |  | .002 | .000 | .000 | .000 | - | - |
| T6SS1 | Correlation Coefficient | .609^**^ | .721^**^ | .250^**^ |  | .721^**^ | .175^*^ | .175^*^ | - | - |
|  | Sig. (2-tailed) | .000 | .000 | .002 |  | .000 | .029 | .029 | - | - |
| *tdh* +  *trh* – | Correlation Coefficient | .884^**^ | 1.000^**^ | .346^**^ | .721^**^ |  | .243^**^ | .243^**^ | - | - |
|  | Sig. (2-tailed) | .000 |  | .000 | .000 |  | .002 | .002 | - | - |
| *tdh* –  *trh* + | Correlation Coefficient | .125 | .243^**^ | .700^**^ | .175^*^ | .243^**^ |  | -.020 | - | - |
|  | Sig. (2-tailed) | .119 | .002 | .000 | .029 | .002 |  | .808 | - | - |
| *tdh* +  *trh* + | Correlation Coefficient | .231^**^ | .243^**^ | .700^**^ | .175^*^ | .243^**^ | -.020 |  | - | - |
|  | Sig. (2-tailed) | .004 | .002 | .000 | .029 | .002 | .808 |  |  | - |
| T3SS1 | Correlation Coefficient | - | - | - | - | - | - | - |  |  |
|  | Sig. (2-tailed) | - | - | - | - | - | - | - | - |  |
| T6SS2 | Correlation Coefficient | - | - | - | - | - | - | - | - | - |
|  | Sig. (2-tailed) | - | - | - | - | - | - | - | - | - |

**. Correlation is significant at the 0.01 level (2-tailed). *. Correlation is significant at the 0.05 level (2-tailed).

A P-value of < 0.05 was considered statistically significant, and P < 0.01 was deemed to be highly significant.
